# T-tubule remodeling in human hypertrophic cardiomyopathy

**DOI:** 10.1007/s10974-020-09591-6

**Published:** 2020-11-22

**Authors:** Giulia Vitale, Raffaele Coppini, Chiara Tesi, Corrado Poggesi, Leonardo Sacconi, Cecilia Ferrantini

**Affiliations:** 1 Department of Experimental and Clinical Medicine, University of Florence, Florence, Italy; 2 European Laboratory for Nonlinear Spectroscopy (LENS), University of Florence, Sesto Fiorentino, Italy; 3 Department NeuroFarBa, University of Florence, Florence, Italy; 4 National Institute of Optics, National Research Council, Florence, Italy

**Keywords:** Hypertrophic cardiomyopathy, T-tubules, Excitation–contraction coupling

## Abstract

The highly organized transverse T-tubule membrane system represents the ultrastructural substrate for excitation–contraction coupling in ventricular myocytes. While the architecture and function of T-tubules have been well described in animal models, there is limited morpho-functional data on T-tubules in human myocardium. Hypertrophic cardiomyopathy (HCM) is a primary disease of the heart muscle, characterized by different clinical presentations at the various stages of its progression. Most HCM patients, indeed, show a compensated hypertrophic disease (“non-failing hypertrophic phase”), with preserved left ventricular function, and only a small subset of individuals evolves into heart failure (“end stage HCM”). In terms of T-tubule remodeling, the “end-stage” disease does not differ from other forms of heart failure. In this review we aim to recapitulate the main structural features of T-tubules during the “non-failing hypertrophic stage” of human HCM by revisiting data obtained from human myectomy samples. Moreover, by comparing pathological changes observed in myectomy samples with those introduced by acute (experimentally induced) detubulation, we discuss the role of T-tubular disruption as a part of the complex excitation–contraction coupling remodeling process that occurs during disease progression. Lastly, we highlight how T-tubule morpho-functional changes may be related to patient genotype and we discuss the possibility of a primitive remodeling of the T-tubule system in rare HCM forms associated with genes coding for proteins implicated in T-tubule structural integrity, formation and maintenance.

## Introduction

T-tubules are transverse and deep invaginations of the surface sarcolemma running along the Z-line regions in mammalian ventricular myocytes. Functionally, T-tubules guarantee a rapid propagation of the action potential (AP) towards the cardiomyocyte core. The high concentration of key excitation–contraction (E–C) coupling proteins on T-tubule membrane, such as dihydropyridine receptors (DHPRs) and other membrane channels/transporters (Orchard et al. 2009; Pásek et al. 2008; Yang et al. 2002), allows synchronous triggering of Ca^2+^ release from the sarcoplasmic reticulum (SR) across the entire cardiomyocyte, as well as simultaneous activation of all myofibril layers. Studying human or animal model cardiac muscle, structural alterations of T-tubules have been described in several cardiac diseases: chronic heart failure, atrial fibrillation as well as secondary hypertrophy (e.g. aortic stenosis or hypertension), or genetic disorders (Cannell et al. 2006; Coppini et al. 2013; Crocini et al. 2016; Crossman et al. 2011, 2015; Dibb et al. 2009; Ferrantini et al. 2017, 2018; He et al. 2001; Heinzel et al. 2008; Høydal et al. 2018; Kaprielian et al. 2000; Kostin et al. 1998; Lenaerts et al. 2009; Louch et al. 2004; Lyon et al. 2009; Manfra et al. 2017; Maron et al. 1975a, b; Ohler et al. 2009; Schaper et al. 1991; Wei et al. 2010). In all the above conditions the most common remodeling pattern of the T-tubular network is characterized by a reduction in the number of transverse components and T-tubular openings (“mouth”) on the surface sarcolemma, with a global loss of T-tubules periodicity at the Z-discs. A spatial and geometrical rearrangement of the residual T-tubular system with a greater proportion of tubules running in the longitudinal and oblique directions, and an increase in the mean T-tubular diameter were also observed. Interestingly, in animal models of secondary hypertrophy as well as of physiological hypertrophy, hand in hand with increased cell dimensions, T-tubule proliferation and density increase have been described.

While the architecture and function of T-tubules have been well described on animal model hearts, T-tubule morpho-functional data on human cardiac samples are scarce. In particular, some human data are available on Heart Failure (HF) and atrial fibrillation, but little is known on human primary and secondary left ventricular (LV) hypertrophy, including hypertrophic cardiomyopathy (HCM) during the non-failing stage of the disease. HCM is the most prevalent primary disorder of the cardiac muscle, with a prevalence of 1 in 500 worldwide (Maron and Maron 2013). It is characterized by asymmetric LV hypertrophy, unexplained by increased loading conditions or other systemic diseases. About 35–60% of patients with HCM are heterozygous for missense or truncating mutations in genes encoding sarcomeric proteins, the most common being *MYH7* (β-myosin heavy-chain), *MYBPC3* (cardiac myosin-binding protein-C) (Ho et al. 2015) and *TNNT2* (Troponin T) (Coppini et al. 2014; Driest et al. 2003; Thierfelder et al. 1994).

Despite the huge heterogeneity of clinical manifestations, most patients maintained a compensated hypertrophic disease stage (Olivotto et al. 2009) with preserved LV function. Rarely the disease evolves into HF, i.e. “end-stage” HCM, a condition that actually does not differ from other forms of HF. The pathophysiology of HCM relies on the close interplay between the primary effects of the gene mutation, directly causing a dysfunction of the myofilaments, and the secondary maladaptive E–C coupling changes. These  latter changes, along with additional adverse myocardial remodeling processes (e.g. fibrosis, myocardial disarray) progress and aggravate during the course of the disease.

The description of HCM-associated T-tubular remodeling is limited to a few reports on the “end-stage” disease (Lyon et al. 2009; Ohler et al. 2009). Data on mouse models carrying sarcomere mutations (Crocini et al. 2016; Ferrantini et al. 2017) are also available, although with a number of limitations to translate them into human pathology (as highlighted in the rest of this review). In the present work, after recapitulating the differences in T-tubule architecture between human and animal cardiomyocytes (to point out the need of direct studies in human cardiac muscle) and after recalling some general notions on HCM, we focus on the structural features of T-tubules in human HCM by revisiting some data obtained from patient myectomy samples (Coppini et al. 2013; Ferrantini et al. 2018; Maron et al. 1975a, b; unpublished data). Next, we discuss the role of T-tubular disruption in HCM pathogenesis as part of the vast E–C coupling remodeling process and how, in rare cases, the disease may be primarily associated to mutation-driven T-tubular damage.

## T-tubule architecture in animal and human cardiomyocytes

Detailed descriptions of the structural and ultrastructural characteristics of the T-tubular network have been obtained in ventricular cardiomyocytes or myocardium from animal models (Jayasinghe et al. 2012). In rodents and small mammals, T-tubules are deep digitiform invaginations of the sarcolemma plasma membrane located just at the level of Z-lines and are rich of contact points with the SR, forming calcium release units (CRUs). T-tubules at each Z-line repeat with the periodicity of sarcomeres (approximately 2.2 µm in relaxed cardiomyocytes), so that each tubule is located in the middle of two *in series* hemi-sarcomeres. T-tubules are interconnected by longitudinal tubules to constitute a network that is often referred to as “transverse-axial- tubular-system”, or TATS, to emphasize the presence of axial (longitudinal) elements in addition to the transverse ones (Ferrantini et al. 2013; Lindner 1957).

TATS sarcolemmal network, by extending towards the cell interior, guarantees a rapid propagation of the action potential to the cell core, allowing the synchronous and homogeneous activation of CRUs regardless of their location (from the sub-sarcolemmal regions to those closer to the center of the cell).

CRUs are specialized regions of contact between the SR and T-tubules where a large number of ryanodine receptors (RyR2, the main channels that release calcium form the SR) reside. RyR2 are located on the junctional SR membrane (SR terminal cisternae), while on the corresponding t-tubular membrane, voltage-sensitive DHPRs are located and coupled in a rather conserved stoichiometric ratio with RyR2 (4 RyR2 for each DHPR)(Scriven et al. 2000).

**Fig. 1:**
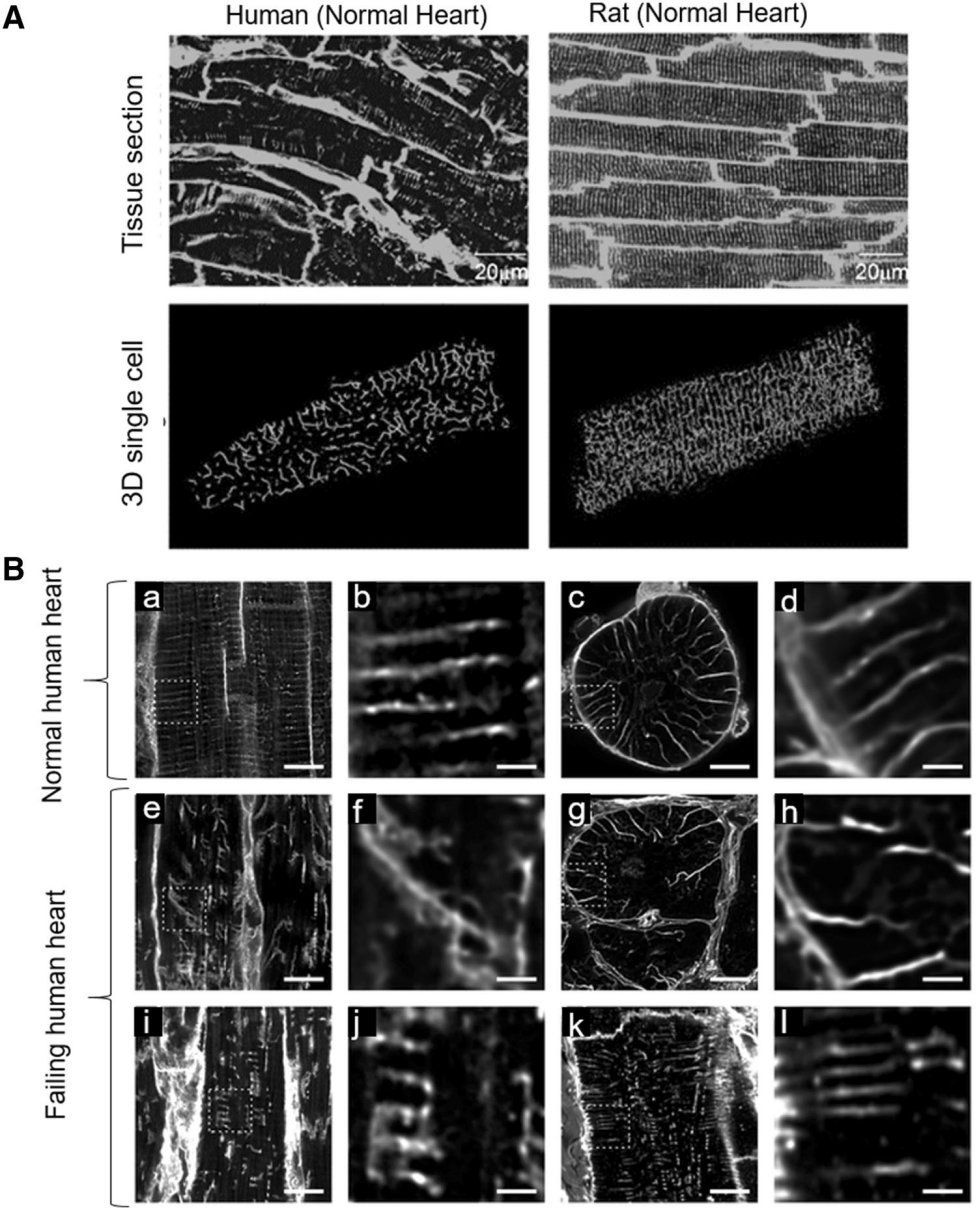
T-tubule organization in human and rodent ventricular myocytes. **A** Confocal images of the T-tubule system in tissue sections from human ventricle (top, left) and rat ventricle (top, right), labeled with wheat germ agglutinin (WGA) and lipophilic membrane indicator FM4-64, respectively. Three dimensional reconstructions of single cardiomyocytes from human and rat ventricle loaded with WGA are shown in the lower panels. Scale bars: 20 µm. **B** WGA labelling of T-tubules in normal and failing human ventricular myocytes. The top row shows images from normal cells in longitudinal and transverse sections (a-d, left to right) and corresponding images from diseased tissue is shown in the lower two rows. (a) Longitudinal sections of normal tissue shows uniformly spaced T-tubules. Occasional axial elements can also be seen. (b) A magnified view of the region shown by the box in a. (c) Normal myocyte in transverse section. A radial ‘‘spoke-like’’ organization of T-tubules is apparent. (d) Enlarged view of the region shown by the box in c. (e, i, k) Longitudinal sections from three different cells from failing heart, demonstrating the range of T-tubular morphologies found in HF with corresponding (f, j, l) magnified views. Note that while the enlarged view in l appears relatively normal, other regions with the same cell (k) are clearly abnormal. (g) Transverse section showing that, while the general direction of diseased tubules is radial, tubules are more disorganized. (h) Magnified view of the region shown by the box in g. Images are projections of 5 slices with z depth of 1 mm. Scale bars in overview images are 10 mm and in close up images 2 mm. HF, heart failure. Reproduced from Manfra et al. (2017) and Crossman et al. (2011)

Clear differences exist in the topology and ultrastructure of the T-tubular network among species. Importantly, the average size of T-tubules (e.g. approximately 200–250 nm in mice and rats, 400 nm in rabbits), the density of T-tubules (e.g. ratio of tubular membrane surface/sarcolemmal membrane surface, < 1 in rabbits, > 1.5 in rodents), the number of T-tubular openings in the sarcolemmal surface (ranging from 1 to 3 million mouths per µm^2^ of membrane surface), as well as the average length of the transverse and axial segments of the network or the presence of narrow/dilated regions, are all features with very large inter-species differences. Small animals with high heart rates at rest (such as mice or rats) require a highly organized and developed T-tubular structure supporting a rapid cycling of Ca^2+^ with high speed of contraction and relaxation. In larger species with lower heart rates, such as pig and dog models, there is a minor need for this complex architecture and, indeed, a great number of cell areas with low density of T-tubules are observed, even in the normal non-diseased hearts (Heinzel et al. 2002). Data on the T-tubule architecture and function can hardly be directly translated form rodents to large mammals or humans. As to human ventricular myocardium, information on T-tubule architecture and function is very scarce, particularly in non-pathological (normal) conditions, because of the scarcity of donor tissue availability. Regional differences within the ventricles (Crossman et al. 2015) that have been described in animal models may also exist in humans, e.g. between LV septum and the free wall, drawing an even more complex picture. The human ventricular myocardium shows a poorly developed T-tubule network, which never reaches the densities and the structural complexity typical of rodent T-tubule system (Fig. 1A). The rodents T-system is highly organized, extensive and geometrically complex with several branching points. In contrast, human T-tubules are fewer and wider with a coarser and more radial arrangement that creates spoke-like structures when observed in transverse section (Jayasinghe et al. 2012). The varying geometries of the T-tubular system may contribute to differences in E–C coupling dynamics among species. Indeed, the reduced complexity of the T-tubule architecture in humans is also reflected by the larger average cross sectional area of contractile myofilaments supplied by each RyR cluster which is larger in humans compared to rats (Jayasinghe et al. 2012).

Without detracting from the value of animal models, these results indicate the importance of studying T-tubules architecture and their potential disease-associated alterations directly on human samples (Fig. 1B).

## Hypertrophic cardiomyopathy: different pathogenic pathways at different stages

**Fig. 2:**
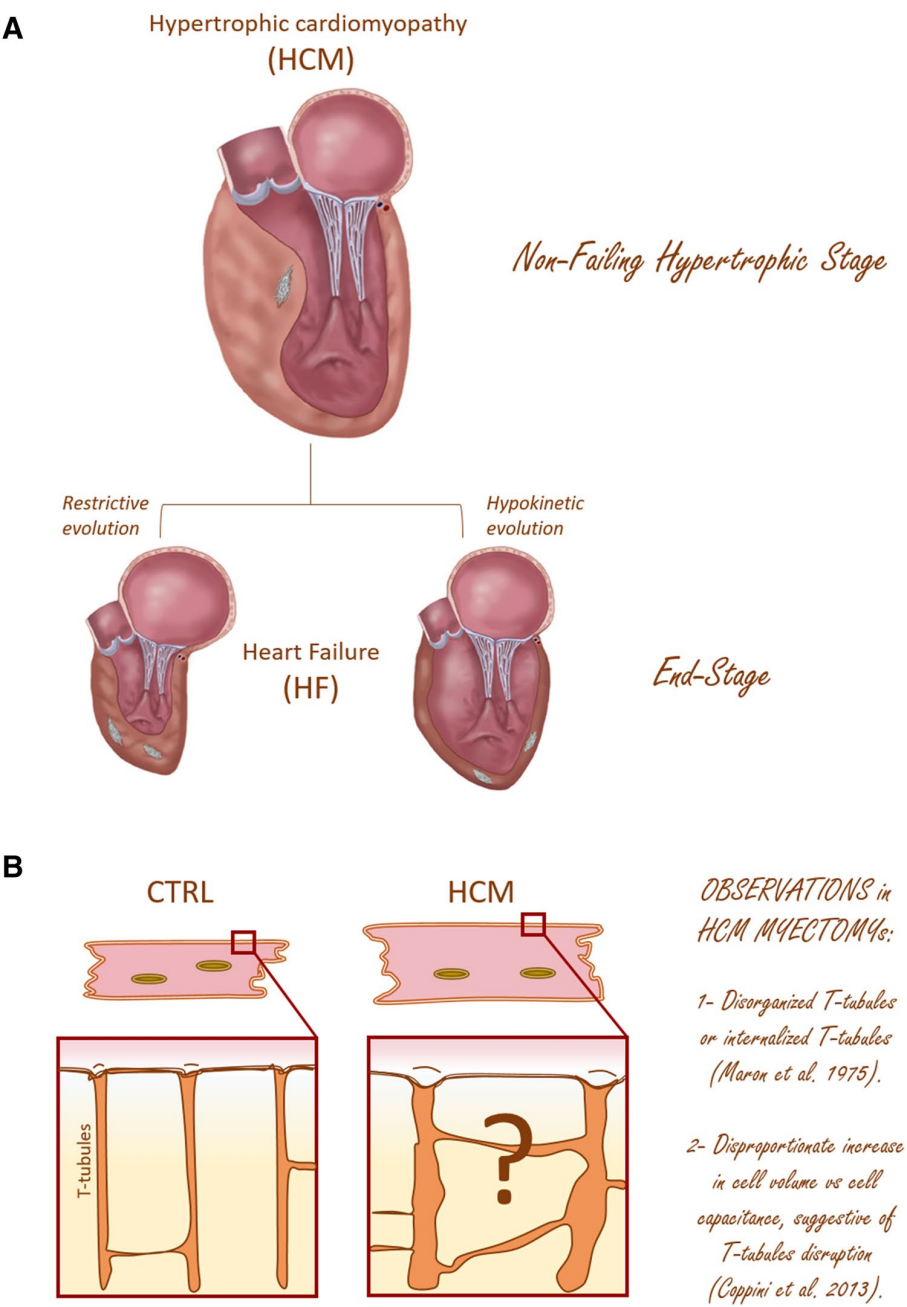
(previous page). HCM: clinical staging and cardiomyocytes remodeling. **A** *Stages of hypertrophic cardiomyopathy from the clinical standpoint.* The pathogenic HCM mutations initiate a life-long remodeling process within the myocardium which presents with distinct clinical disease stages. The “Non-failing hypertrophic stage” which is characterized by an hypertrophied and hyperdinamic LV (with an ejection fraction > 65%). About 75% of HCM patients belong to this class. Importantly, during this stage patients may undergo cardiac surgery, named “myectomy”, to relieve LV outflow obstruction, thus giving the possibility to collect samples for biophysical studies. The “end-stage” condition instead is reached by a small subset of patients (5%). This latter condition is characterized by severe functional deterioration of the LV (defined by an LVEF < 50%), clinical decompensation and terminal HF. Sometimes patients are implanted with a contraction assist device (LVAD) or heart transplanted; these events represent another source of myocardial samples. Modified from Coppini et al. (2014). **B** *HCM versus Normal Heart.* In normal heart, T-tubules are periodically located at the level of Z-lines, and are rich of contact points with the SR, forming calcium release units (CRUs). This organization is crucial in ensuring a homogeneous Ca^2+^-release throughout the cell, thus allowing synchronous myofilaments contraction. In HCM hearts, cardiomyocytes appeared hypertrophied and a structural remodeling of the T-tubular network may be present but data on myoctomy samples are scarce and difficult to collect. Different cell types were coexisting in the same diseased hearts and were classified as hypertrophied but non-degenerated cells or cells with evidence of mild to severe degeneration (Maron et al. 1975a, b)

HCM is a primary progressive disease of the heart muscle that affects one in 500 people and is due in most cases to mutations in sarcomere protein genes, transmitted with a mendelian inheritance (Maron and Maron 2013). Figure 2A represents the stages of the disease from the clinical standpoint (Olivotto et al. 2012). The most common HCM-related phenotype is characterized by an asymmetric hypertrophy of the interventricular septum. The echocardiographic observation of LV hypertrophy, in the absence of hemodynamic determinants (e.g. aortic valve stenosis), often during adolescence or young adulthood, leads to the suspicion of a genetic origin. In addition to septal thickening, clinical symptoms may appear during the hypertrophic stage: dyspnea, palpitations, syncopal episodes, atrial fibrillation, rarely fatal ventricular arrhythmias. This stage of the disease, i.e. the non-failing hypertrophic stage, can persist with a low rate of complications for many years and, only rarely (less than  5% of cases), evolves towards LV dysfunction, clinical decompensation and terminal HF. In these cases, patients show severe HF symptoms and have reached the terminal stage of the disease, defined as “end-stage” HCM (Olivotto et al. 2012) (Fig. 2A).

To the end of this review, we need to distinguish the two disease stages, i.e. the “non-failing hypertrophic stage” and the “end-stage”, as profoundly different and distinct. In both stages HCM patients may undergo cardiac surgery (for different purposes) and myocardial samples may become available for biophysical studies (Fig. 2A). During the hypertrophic non-failing stage, a number of HCM patients undergo surgery to reduce the extent of septal hypertrophy if the thickened upper septum obstructs the outflow of blood during LV ejection. The surgical intervention, namely myectomy, can provide septal myocardial samples to be dedicated to structural or functional studies. This type of samples therefore comes from hearts that have a vigorous mechanical function with preserved (or even increased) ejection fraction. The other possible event when a sample of myocardial tissue may become available for collection and study is when HCM patients are implanted with a contraction assist device (LVAD) or heart transplanted. In this case the sample can derive from different portions of the LV of the failing heart: not necessarily the interventricular septum, but rather the LV free wall. Mechanisms underlying the disease in these two stages are likely profoundly different (Fig. 2B). For instance, in human myomectomy samples (“non-failing hypertrophic stage”) force amplitude and frequency dependency of twitch contractions are preserved while they are impaired in end stage HCM and HF (Lyon et al. 2009).

**Fig. 3:**
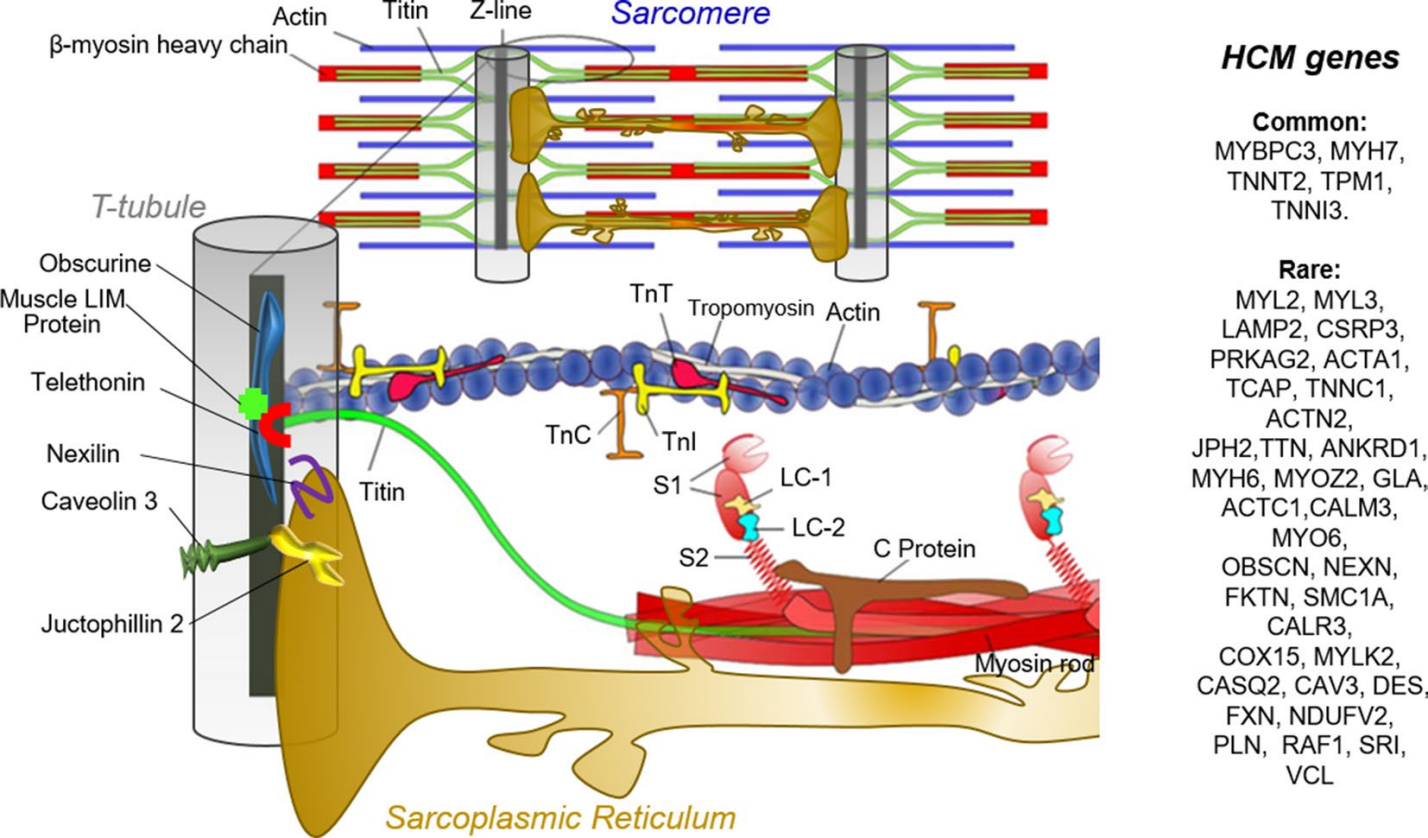
Gene mutations associated to HCM. Cartoon depicting the sarcomeres and the associated T-tubule sarcoplasmic reticulum structures. About 35–60% of patients with HCM are heterozygous for missense or truncating mutations in genes encoding sarcomeric proteins, with the most commonly involved being MYBPC3 (cardiac myosin-binding protein-C), MYH7 (β-myosin heavy-chain), and TNNT2 (Troponin T) or TPM1 (Tropomyosin). Rare forms of HCM (prevalence < 1%) are those associated to other genes that are listed on the right panel. Among them, additional sarcomere proteins and Z-line proteins, e.g. TnC, Troponin C; TnI, Troponin I, LC, light chain; TTN, Titin, OBSCN, Obscurine; or proteins involved in E–C coupling and muscle regulation/development (JPH2, Junctophillin 2; CAV3, Caveolin-3; CSRP3, Muscle LIM Protein; NEXN, Nexilin; TCAP, Telethonin)

Whether and when T-tubule structural alterations appear in HCM progression as part of the secondary E–C coupling remodeling process still need to be elucidated. In fact, among common and certainly pathogenic HCM mutations (Fig. 3) we find sarcomeric proteins involved or closely associated to the motor function and its calcium regulation (β-myosin heavy chain, myosin-binding protein C, troponin T and tropomyosin). These mutations are responsible for a series of primitive changes in myofilament function, i.e. altered crossbridge mechanics, cycling kinetics, and energetics (Belus et al. 2008; Ferrantini et al. 2009; Robinson et al. 2007; Spudich 2019; Toepfer et al. 2020), or impaired switched-off state of the thin filament at low [Ca^2+^] (Tardiff et al. 2015). Hand in hand with the disease progression, these primitive changes are accompanied by a number of E–C coupling and myofilament post-translational modifications and activation of remodeling pathways, partially in common with those of secondary hypertrophy and heart failure. The loss of T-tubules, if present, resides in the number of “acquired” alterations and participate to a complex secondary remodeling process that involves both cellular electrophysiology (e.g. changes in several transmembrane ion currents), alterations of intracellular Ca^2+^ handling (e.g. Ca^2+^ transient kinetics and diastolic Ca^2+^ levels) (Coppini et al. 2013, 2017; Ferrantini et al. 2017, 2018) as well as remodeling of the extracellular matrix (Ariga et al. 2019) and fibrosis. Figure 3 also shows that a number of genes coding for T-tubule associated proteins either implicated in calcium homeostasis or in T-tubule formation have been recently associated to rare forms of HCM (e.g. Junctophillin, Caveolin, etc.), see also Table 2. In these cases, T-tubule disruption may be a direct primitive consequence of the disease-causing mutation as will be discussed at the end of this review.

## T-tubules in HCM

Profound remodeling of the T-tubular network has been described in terminal HF, both in animal models and in humans (Crossman et al. 2011; He et al. 2001; Høydal et al. 2018; Louch et al. 2004; Lyon et al. 2009). Human samples have been derived from patients who had undergone LVAD implantation or cardiac transplantation because of terminal HF of various etiology. i.e., acute or chronic ischemic disease, valvulopathies, dilated cardiomyopathy, but also HCM (Table 1). Established features of HF-associated T-tubule remodeling are the following: reduction of the transverse T-tubular elements with an increase in the longitudinal components, decreased number of T-tubular mouths on the cell surface, presence of dilated tubules, and loss of localization of T-tubules with respect to the Z-lines, so that the T-tubule is "hanging" towards one hemi-sarcomere (Cannell et al. 2006; Coppini et al. 2013; Crocini et al. 2016; Crossman et al. 2011, 2015; Dibb et al. 2009; Ferrantini et al. 2017, 2018; He et al. 2001; Heinzel et al. 2008; Høydal et al. 2018; Kaprielian et al. 2000; Kostin et al. 1998; Lenaerts et al. 2009; Louch et al. 2004; Lyon et al. 2009; Manfra et al. 2017; Maron et al. 1975a, b; Ohler et al. 2009; Schaper et al. 1991; Wei et al. 2010).

**Table 1:** T-tubules in human Left Ventricular samples

Year	Disease	Samples studied	Methods	Findings on T-tubule remodelling	References
1975	**HCM (Non-failing hypertrophic stage)** and LV hypertrophy of varied causes (i.e. aortic stenosis)	Fixed LV or ventricular septum biopsy samples	EM	Irregularly shaped or dilated T-tubules in hypertrophied cells; loss of T-tubules in degenerating cells	Maron et al. (1975a)
1975	LV hypertrophy in patients with chronic aortic valve disease	Fixed LV or ventricular septum biopsy samples	EM and light microscope	Decreased or absent T-tubules; dilatation	Maron et al. (1975b)
1991	End-stage DCM	Fixed LV tissues (frozen sections)	EM	Numerous, dilated T-tubules in hypertrophied, or T-tubule loss in degenerative cells	Schaper et al. (1991)
1998	End-stage DCM	Fixed LV tissues (frozen sections)	EM/Confocal immunofluoresence	T-tubule dilation	Kostin et al. (1998)
2000	End-stage DCM/ICM	Frozen LV tissues	EM/confocal immunofluorescence	Increase in size and number of T-tubules. Increased number of longitudinal elements	Kaprielian et al. (2000)
2009	**HCM (End-stage)**, DCM and ICM	Isolated myocytes from human HF hearts	Confocal microscope with membrane selective dye and ion conductance microscope	Loss of T-tubule openings; decrease in T-tubule density	Lyon et al. (2009)
2009	**HCM (End-stage)**, DCM and ICM	Isolated LV myocytes	Two-photon microscope with membrane selective dye	Only small, but not significant changes in T-tubule network	Ohler et al. (2009)
2011	End-stage DCM	Fixed, frozen LV tissues	Confocal microscope with membrane selective dye	Reduction in orderly pattern, less uniform with more transverse components; dilation	Crossman et al. (2011)
2013	**HCM (Non-failing hypertrophic stage)**	Fresh myectomy samples, single isolated septal cardiomycytes	Cell capacitance/cell valume ratio	Reduction of T-tubular vs surface sarcolemmal membrane area	Coppini et al. (2013)
2017	**HCM (Non-failing hypertrophic stage)**	Fresh myectomy samples, single isolated septal cardiomyocytes	Confocal microscope with membrane selective dye	Low density or negligible presence of T-tubules	Ferrantini et al. (2018)
2018	Post-myocardial infarction HF	Isolated myocytes from human HF hearts	Confocal microscope with membrane selective dye	T-tubule disorganization and loss	Høydal et al. (2018)

In humans, early reports based on histological examinations in failing heart tissue sections showed T-tubular dilation with either increased (Wong et al. 2001) or decreased (Kaprielian et al. 2000; Kostin et al. 1998) density of T-tubules, while in explanted hearts no significant T-tubules loss compared to isolated cells was detected (Louch et al. 2004). These contrasting observations left open the question of whether low T-tubule density was failure-related or normal features of healthy human myocardium

In a recent study Crossman and coworkers, showed that the regions with poor contractile performance have a different T-tubule structure than regions with stronger contraction in failing human hearts, hypothesizing that the variability in the reported extent of T-tubule remodeling in human HF might rely on a sampling problem (Crossman et al. 2015)

Indeed, earlier studies confirmed, through a standard quantification of T-tubular density with di-8-ANEPPS surface staining, that in failing human myocardium T-tubules density was two to three times lower compared to healthy donor cardiac muscle (Cannell et al. 2006; Lyon et al. 2009)

In addition, detailed topographic images of live myocytes detected using a scanning ion conductance microscopy (SICM) (Miragoli et al. 2011) confirmed the loss of T-tubular invaginations in ventricular myocytes from HF human hearts (Lyon et al. 2009). There are a few reports regarding the structure and function of T-tubules in human diseases other than terminal heart failure. In a recent work (Lyon et al. 2009), T-tubule changes were seen in myocytes from end-stage HCM patients. Hoydal and coworkers, first showd in human myocardium that T-tubule disorganization and loss are present earlier before setting of failing conditions, in early stage of human post-myocardial infarction HF (Høydal et al. 2018)

*EM* electron microscopy; *DCM* dilated cardiomyopathy; *HCM* hypertrophic cardiomyopathy; *HF* heart failure; *ICD* ischaemic cardiomyopathy; *LV* left ventricle

Modifyed and up-dated from "Emerging mechanisms of T-tubule remodelling in heart failure" Guo et al. (2013)

A number of papers show that end-stage HCM does not differ from other forms of terminal HF in terms of T-tubule disruption (Table 1). Information about the non-failing hypertrophic phase of the disease, obtained from myectomy samples, is instead poor (Table 1, Fig. 2B). One reason is that HCM samples derived from myectomies should be compared with septal myocardium from non-failing non-hypertrophic patients or non-transplanted donor hearts but these types of samples are rare. Importantly, T-tubule remodeling should always be considered in parallel with the available information on cell size. In fact, T-tubules simply “extend” the cell surface. In HCM, as well as in any type of compensated or non-compensated forms of LV hypertrophy (ranging from the physiologic exercise hypertrophy to the pathologic forms), cellular hypertrophy is the main mechanism of LV mass increase (hyperplastic growth in the heart is negligible): the T-tubules may or may not "keep up" with cell growth. In physiologic, exercise related hypertrophy, cellular hypertrophy is associated with a proliferation of the tubular system, as described in animal models (Kemi et al. 2011). In the case of pathologic secondary LV mass increase (e.g. in hypertension, chronic aortic valve disease or other valve defects), cell volume and cell surface growth are not proportionate, and the relative reduction of cell surface area occurs entirely at the expenses of the T-tubular component.

In 1975, Maron et al. first described myocardial ultrastructure in ventricular samples from patients with HCM as well as secondary forms of LV hypertrophy (i.e. chronic aortic valve disease, alone or in combination with mitral rigurgitation) (Maron et al. 1975a, b). Based on light and electron microscope (EM) observations, made on surgical LV biopsies, they identified various cardiac myocyte typologies, according to the nature and the extent of the morphologic changes shown. Different cell types were coexisting in the same hearts and were classified as hypertrophied non-degenerated cells or cardiac muscle cells with evidence of mild to severe degeneration (Fig. 2B). Importantly, hand in hand with the progression of cardiomyocytes’ morphological degeneration, they observed an aggravation of T-tubule remodeling. Specifically, in each EM section the authors highlight: (a) *hypertrophied but non-degenerated cells:* cardiomyocytes with markedly increased cell volume and irregularly shaped, often dilated, T-tubules; (b)* moderately degenerated cells:* cardiomyocytes with normal cell volume, shallow plasma membrane invaginations, not related to Z-bands, and rare discrete T-tubules; (c) *severely degenerated cells:* cardiomyocytes with reduced cell volume and no discrete T-tubules but large and shallow membrane invaginations disconnected from the cell surface. These “disconnected invaginations”, i.e. internalized T-tubules that resemble vacuoles, are irregularly distributed and do not have any spatial relation to myofibrils at the Z-bands. They probably represent the final stage of the dilatation and disorganization process that T tubules can undergo. The first type of cells (hypertrophied but non-degenerated cells) were present in HCM but also in secondary forms of LV hypertrophy or combined valvular defects. Moderately to severely degenerated muscle cells, while present in HCM patients or patients with combined valvular defects, were instead not observed in patients with predominant aortic stenosis.

**Fig. 4:**
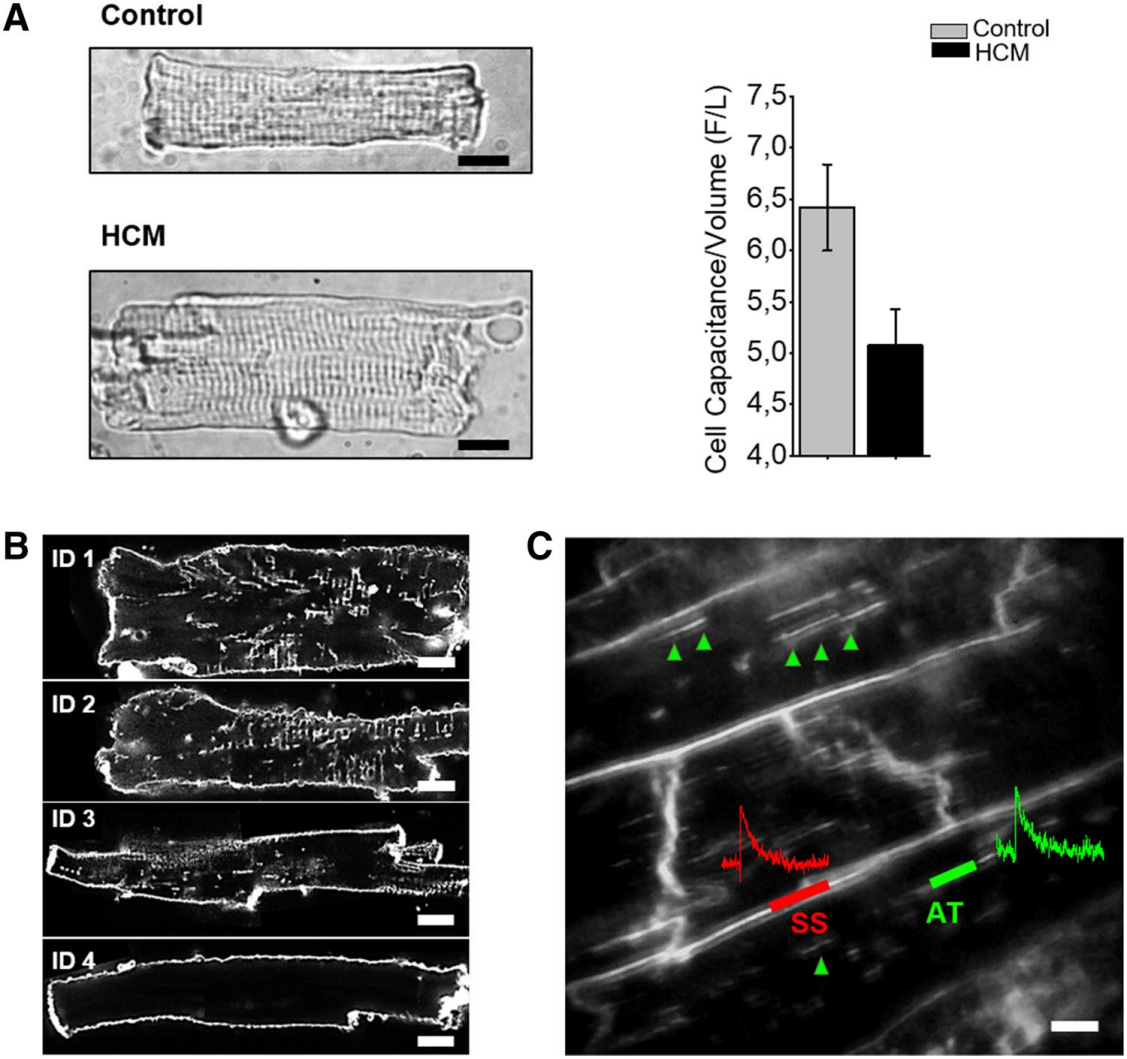
T-tubule remodeling in human HCM myectomies. **A** Left: Representative images of a control (top) and an HCM (bottom) cardiomyocyte, showing cell hypertrophy in HCM. Right: Surface/volume ratio in HCM and control cardiomyocytes; surface is derived from cell capacitance, volume estimated from cell area. Data from 64 cells (14 patients). From Coppini et al. (2018). **B** The density of T-tubules is markedly low in HCM cardiomyocytes. Representative confocal images of single cardiomyocytes. Each cell derives from a different HCM patient sample (ID of the patient is indicated next to the cell in each respective image). Cells were stained with Di-3ANEPPDHQ (Thermo-Fisher) and imaged with a Leica Confocal microscope using the 488 nm laser line. Sections were taken at mid cell. While the outer sarcolemma is well stained in all myocytes, T-tubules are barely visible in most of them and some cells are completely devoid of T-tubules. White bars equal 10 μm. Modified from Ferrantini et al. (2018). **C** Loss of transverse tubules and functionality of axial components in human HCM cardiomyocytes. Two photon fluorescence image of one Di-4-AN(F)EPPTEA labelled HCM trabecula from the left ventricle. The lines mark the probed sarcolemmal regions: surface sarcolemma (SS) in red and axial tubules (AT) in green. White bars equal 10 μm

In the five-year period between 2008 and 2013, we collected myocardial tissue form 26 HCM myectomy patients, the large majority of them carrying sarcomeric mutations, and 4 non-hypertrophic non-failing controls. In HCM cardiomyocytes we showed a significant increase in cell size, estimated from video-microscopy cell surface measurements. This increase was not accompanied by a commensurate increase in cell capacitance, as measured from the same cells in patch clamp experiments (Coppini et al. 2013) (Fig. 4A). As cell capacitance is directly proportional to sarcolemma extension, it represents an extremely reliable index of how large the cell surface is. Specifically, in all hypertrophied HCM myocytes that were tested, the ratio between cell capacitance and cell volume was reduced compared to control cardiomyocytes (5.08 ± 0.35 F/L vs. 6.42 ± 0.42 F/L respectively, P < 0.05), reflecting a disproportion between surface vs. volume growth (Coppini et al. 2013; Coppini et al. 2018). The reduced cell capacitance/cell volume ratio in HCM myocytes is a strong indication of a disrupted T-tubular network. Images obtained with the confocal microscope (Ferrantini et al. 2017) from the same HCM cardiomyocytes labelled with a membrane fluorescent dye, somehow reproduced the variability in cell size and T-tubule architecture observed by Maron et al. in EM studies (Fig. 4B). Along with a majority of hypertrophic cells with largely increased cell volume and irregularly shaped T-tubules (Fig. 4B, ID1–2), we also found cells with normal to reduced cell volume and rare discrete T-tubules (Fig. 4B, ID3–4). Membrane selective fluorescent dyes that are sensitive to voltage variations (voltage-sensitive dyes, VSD) can be employed to monitor the electrical activity of T-tubules still connected to the surface. In this regard, one example of AP recordings from myectomy tissue is reported in Fig. 4C (unpublished data). The measurements were obtained using a random-access multiphoton (RAMP) microscope (Iyer et al. 2006) in combination with fluorinated VSD (Yan et al. 2012), that allowed us to simultaneously measure the AP at surface sarcolemma and surface-connected T-tubules, in neighboring cardiomyocytes within the myectomy tissue (Ferrantini et al. 2014; Sacconi et al. 2012). We observed that the irregularly shaped T-tubules, either running in transverse or longitudinal directions, were still able to conduct the AP.

This observation cannot be taken for granted. In fact, we had previously demonstrated that the mere presence of T-tubules does not ensure its electrical function (Sacconi et al. 2012): T-tubules structurally coupled to the surface sarcolemma occasionally fail to conduct the AP (electrical uncoupling) and are thus associated with impaired local Ca^2+^ release (Crocini et al. 2014).

RAMP microscopy may also be used to record simultaneously the electrical activity of T-tubules and the correspondent local Ca^2+^ release (Crocini et al. 2014, 2016; Sacconi et al. 2012). With this configuration isolated cardiomyocytes are stained with a fluorescent Ca^2+^ probe (e.g. FluoForte GFPcertified), and a VSD (e.g.di-4-AN(F)EPPTEA), that are simultaneously excited (Crocini et al. 2016). With this approach, we demonstrated the existence of failing T-tubules i.e, tubules that do not conduct AP and are associated to delayed Ca^2+^ release in HF as well as in HCM animal models (Crocini et al. 2016; Sacconi et al. 2012; Scardigli et al. 2017).

**Fig. 5:**
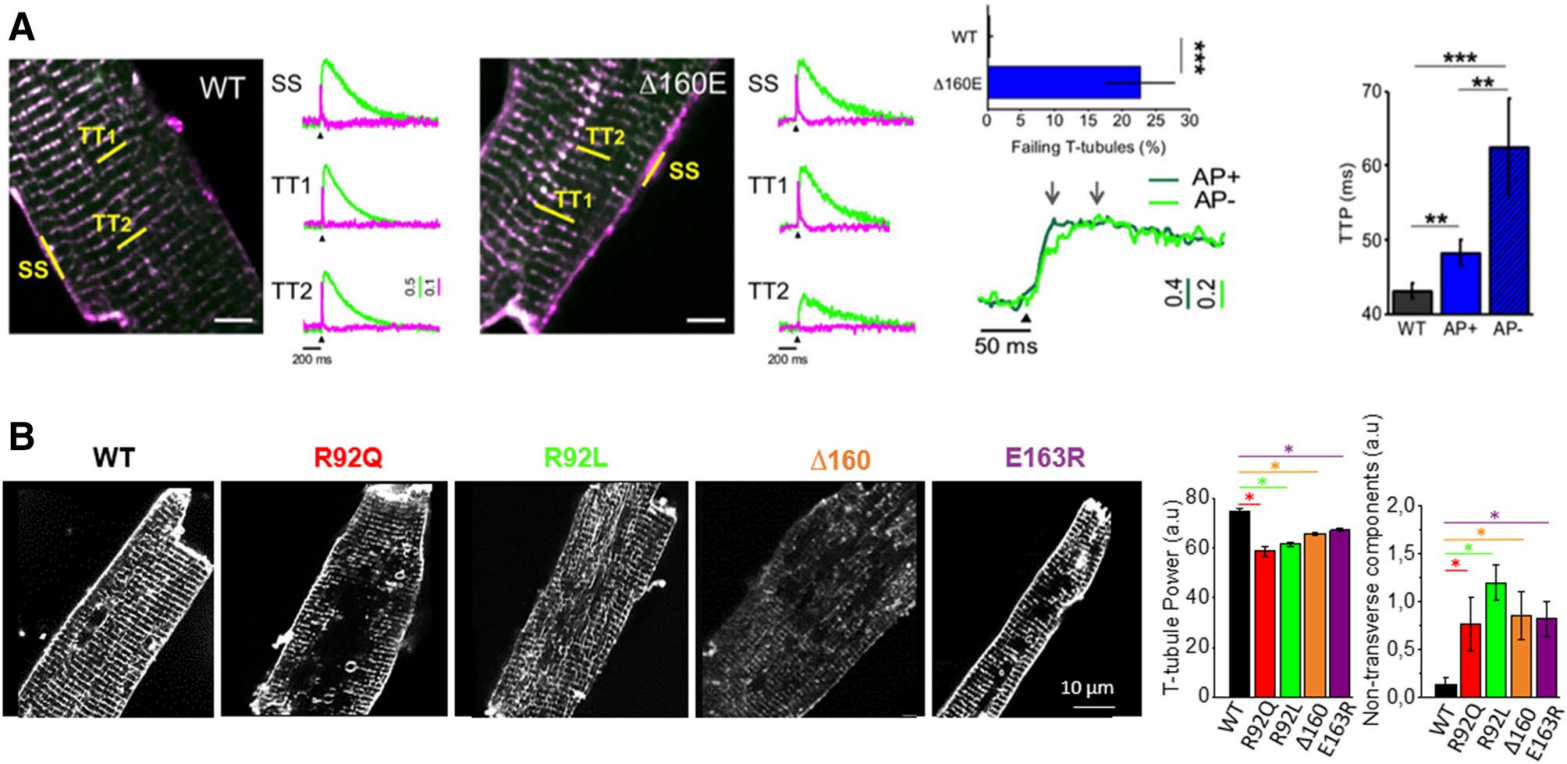
Alterations of T-tubules in mouse models of HCM. **A** Defects of T-tubules electrical activity and local calcium release in cTnT Δ160E mouse model. Left: two-photon fluorescence (TPF) image of a stained cTnT Δ160E and a WT ventricular myocyte: sarcolemma in magenta (di-4-AN(F)EPPTEA) and [Ca^2+^]_i_ in green (GFP-certified Fluoforte). Scale bar in white: 5 μm. Right: representative normalized fluorescence traces (ΔF/F0) of SS and two T-tubules (TTi) recorded in WT and cTnT Δ160E cardiomyocyte (average of ten subsequent trials). Membrane potential in magenta, [Ca^2+^]_i_ in green. AP elicited at 200 ms (black arrowheads). Middle: (top) Columns showing the percentage of electrically failing T-tubules in WT and cTnT Δ160E myocytes. Data from 101 WT and 66 cTnT Δ160E T-tubules (Student’s t-test ***p b 0.001). (bottom) Superposition of fluorescence Ca^2+^ traces (ΔF/F0) of electrically coupled (AP+, dark green) and uncoupled (AP−, green) T-tubules reported above. The two grey arrows pinpoint Ca^2+^ transients TTP of the traces. Electrical trigger provided at 200 ms (black arrowhead). (right) Columns showing time-to-peak (TTP) mean values of Ca^2+^ release measured in cTnT Δ160E cells with respect to WT. Ca^2+^ transient kinetics is reported by separately analysing the two populations of T-tubules (AP+ and AP−). Data reported as mean ± SEM from 101 WT T-tubules, 65 AP+, and 15 AP− (n = 28 WT and 17 cTnT Δ160E; N = 10WT and 7 cTnT Δ160E). Student’s t-test **p b 0.01, ***p b 0.001. Modified from Crocini et al. (2016). **B** Left: Representative confocal images from isolated LV cardiomyocytes stained with di-3-aneppdhq from WT, R92Q, R92L, ∆160 and E163R hearts. Horizontal bar equals 10 µm. Right: Columns showing T-tubule Power, as calculated using the TTorg ImageJ plugin, and non-transverse components in cardiomyocytes from the five cohorts of mice. Means ± S.E. Modified Statistics: One-way ANOVA with Tukey correction.*P < 0.05

In details, as shown in Fig. 5A, a well-established HCM mouse model harboring the Δ160 cardiac troponin T (cTnT) mutation was employed to characterize the morpho-functional features of the tubular system in comparison to WT cardiomyocytes. Although not markedly altered in structure, the tubular system of cTnT-Δ160 HCM cardiomyocytes did not adequately conduct the action potential, with high occurrence of AP-propagation failure episodes. More than 20% of T-tubules failed in propagating APs with the associated junctional regions displaying a significantly delayed local Ca^2+^ release (Crocini et al. 2016). Functionally, CRUs that are coupled to failing T-tubules behave exactly as the “orphaned” CRUs, i.e. the RyR2 clusters that are no longer structurally coupled with a T-tubule (Gómez et al. 2001; Song et al. 2006). A link may then exist between some specific mutation and the development of T-tubule morpho-functional alterations, including the potential occurrence of AP-failures. As an example of the potential role of genetic factors in driving T-tubule remodeling, we report images and structural data from three additional HCM mouse models, harboring different cTnT mutations (R92Q, R92L, E163R,) (Fig. 5B). Of note, all these cTnT mouse models, tested at 6–8 months, show preserved ejection fraction and cardiac output, well reproducing the Non-failing Hypertrophic stage of the human disease. Compared to WT, low density of transverse tubules and excess of longitudinal and tangled T-tubules can be observed in the cTnT mutants. Notably, mutants with different TnT mutations (even within the same coding gene, R92Q vs R92L), showed a variable reduction of tubular transverse components and a variable increase in longitudinal elements, suggesting a genotype-driven remodeling of the T-tubule network. At variance with the cTnT-Δ160 HCM the other mutants have not yet been characterized in terms of AP failure occurrence. The link between HCM genotype and T-tubule remodeling is at the moment rather obscure and calls for future studies of the morpho-functional characteristics of the T-tubular network in a large group of myectomy samples, classified according to the patient's genotype.

## Role of T-tubular disruption in HCM phenotype: non-homogeneous calcium activation

The functional role of T-tubular remodeling within the complex electrophysiological and E–C coupling alterations observed in human HCM myectomy samples needs a careful contextualization. Compared with controls, HCM cardiomyocytes showed prolonged APs related to increased late Na^+^ (*I*_NaL_) and Ca^2+^ (*I*_CaL_) currents and decreased repolarizing K^+^ currents, increased occurrence of cellular arrhythmias, prolonged Ca_i_^2+^ transients, and higher diastolic intracellular Ca^2+^. Such changes were related to enhanced Ca^2+^/calmodulin kinase II (CaMKII) activity and increased phosphorylation of its targets as well as variations in SR proteins expression and function (e.g. decreased SERCA and increased RyR2 activity) (Coppini et al. 2013; Schotten et al. 1999). In contrast to failing human or end-stage human HCM myocardium, measurements of active tension in intact HCM trabeculae dissected from the endocardial layer of the myectomies showed a positive force-frequency relationship and a preserved contractile reserve (under isoproterenol or high external calcium), in agreement with the maintained Ca_i_^2+^ transient amplitude and SR Ca^2+^ load observed in HCM cardiomyocytes from the same samples (Coppini et al. 2013).

At first glance, these changes in calcium handling and cellular electrophysiology (summarized in Table 3) have little to do with the changes in EC coupling promoted by T-tubule disconnection. The use of an osmotic shock protocol, first developed in single cardiac cells (Kawai et al. 1999) and later adapted to intact trabeculae (Ferrantini et al. 2014), has provided significant information about the impact of “pure” T-tubule disconnection, namely “acute detubulation”, in the absence of other disease-driven modifications. The main electrophysiological and mechanical effects of “acute detubulation” are reported in Table 3. In brief, acute T-tubule disconnection causes a shortening of the AP with a marked decrease of *I*_CaL_ (preferentially located at the T-tubules) (Brette et al. 2002; Ferrantini et al. 2014; Kawai et al. 1999) but no changes in *I*_NaL_ or repolarizing K^+^ currents (ubiquitariously distributed in the sarcolemma)(Yang et al. 2002), no variations in the occurrence of cellular arrhythmias, no variations in SR Ca^2+^ load or diastolic Ca^2+^_i_ but reduced amplitude and prolonged duration of Ca^2+^ transients (Brette et al. 2005; Ferrantini et al. 2014). In analogy to failing human or end-stage human HCM myocardium, measurements of active tension in intact acutely detubulated trabeculae showed an impairment of the force-frequency relationship (Ferrantini et al. 2014). A point by point comparison between pathologic changes observed in HCM and HF and modifications related to “acute detubulation” is proposed in Table 3 to highlight how the structural and functional remodeling of membrane channels and Ca^2+^ handling in HCM cardiomyocytes is profoundly different from what expected as a direct effect of T-tubule disconnection. The only “matching” observations are the prolonged time course of Ca^2+^ transients and twitches. Non-uniform calcium induced calcium release associated with “detubulation” may be an important pathogenic mechanism in HCM cardiomyocytes. The inhomogeneity of calcium release may lead to delayed activation of some CRUs (“orphaned” or associated with non-functioning T-tubules) and thus to delayed rising time (time to peak) of the global calcium transients. This mechanism of alteration of the Ca^2+^ transient time course in HCM is not the only one as also reduced SERCA function and changes in myofilament Ca^2+^ sensitivity have been reported to occur in HCM (Coppini et al. 2013; Robinson et al. 2007; Schotten et al. 1999). However, the T-tubule mechanism is likely the most relevant to explain the delay in the rising phase of the calcium transients. The inhomogeneity and spatio-temporal dissynchrony of calcium release would also lead to the inhomogeneity in the activation of adjacent sarcomeres, triggering abnormal inter-sarcomeric dynamics that may further slow-down the speed of force development. This may help to explain the delayed time to peak of contraction observed in twitching human HCM trabeculae, an observation that otherwise would remain unexplained. Non-uniform calcium release, indeed, can also promote the initiation of propagated calcium waves, induce beat-to-beat and regional variability of AP duration and, in general, promote arrhythmias, especially under conditions of SR and cytosolic Ca^2+^ overload, which are observed in HCM cardiomyocytes (Coppini et al. 2013).

## Primary remodeling of T-tubules in rare forms of HCM

Apart from sarcomeric HCM, independent studies have recently identified rare genetic mutations (that account for less than 1% of cases) in genes coding for Ca^2+^ handling, Z-disc or cytoskeleton proteins (Bos et al. 2006; Hayashi et al. 2004a, b; Landstrom et al. 2007; Wang et al. 2010; Xu et al. 2015) that are pathogenic for HCM. A list of these genes and their association with HCM and/or other forms of cardiomyopathy is shown in Table 2. Of note, these proteins have been shown to be involved in T-tubule formation, cycling, function and stabilization, e.g. junctophilin 2, caveolin-3, amphyphisin-2 (Bin1), telethonin (Tcap), etc.

**Table 2:** Proteins involved in T-tubule regulation

Gene	Protein	Protein role/function	Association to HCM	Association to other cardiomyopathies	References
*JPH2*	Junctophilin-2	Membrane-binding protein critical for accurate association of T-tubule and junctional SR membrane; it has regulatory functions on local ion channels and intracellular Ca^2+^ signalling; it provides an anchor for developing T-tubules during maturation of cardiac Ca^2+^ handling	Yes	Yes, DCM	Beavers et al. (2014); Chen et al. (2012); Jones et al. (2019); Landstrom et al. (2011); Landstrom et al. (2007); Matsushita et al. (2007); Reynolds et al. (2016); van Oort et al. (2011); Wei et al. (2010)
*BIN-1*	Amphiphysin 2	Membrane deforming protein which contributes to membrane trafficking and remodeling, cytoskeleton dynamics, DNA repair, cell cycle progression, and apoptosis; essential for T-tubule biogenesis being a main factor in inducing membrane invaginations; required for trafficking and clustering LTCC into t-tubules and recruiting phosphorylated RyRs for coupling with LTCCs	no	Yes, DCM	Hong et al. (2012); Hong et al. (2010); Hong et al. (2014); Laury-Kleintop et al. (2015); Lyon et al. (2009); Muller et al. (2003); Prokic et al. (2014)
*CAV3*	Caveolin-3	Structural protein of caveolae in muscle; involved in the biogenesis of the T-tubule system; and trafficking LTCC regulatory proteins and I_Ca_ to the t-tubules	Yes	Yes, DCM	Catteruccia et al. (2009), Galbiati et al. (2001), Hayashi et al. (2004b), Traverso et al. (2008)
*NEXN*	Nexilin	Pivotal protein component of the junctional membrane complex; it is required for Z-disk stabilization and overall T-tubule formation	Yes	Yes, DCM	Hassel et al. (2009), Wang et al. (2010)
*TCAP*	Telethonin	Stretch-sensitive Z-disc protein that binds to proteins in the T-tubule membrane; essential for load-dependent formation of T-tubules in striated muscle; it may constitute a mechano-electrical links between Z-lines and T-tubules	Yes	Yes, DCM	Hayashi et al. (2004a), Ibrahim et al. (2013), Knöll et al. (2002)
*OBSCN*	Obscurin	Structural protein required for the organization of myofibrils during sarcomere assembly	Yes	Yes, DCM and LV non-compaction cardiomyopathy	Marston et al. (2015), Raeker et al. (2006), Rowland et al. (2016), Xu et al. (2015)
*TTN*	Titin	Giant protein that anchors in the Z-disc and extends to the M-line region of the sarcomere; it acts as a molecular spring that maintains the precise structural arrangement of thick and thin filaments, and gives rise to passive muscle stiffness; the titin–telethonin complex is somehow implicated in the organization or maintenance of T-tubules near the Z-disk	Yes	Yes, DCM	Bos et al. (2006), Hayashi et al. (2004a), Itoh-Satoh et al. (2002)
*DYSF*	Dysferlin	Protein involved in membrane repair, vesicle fusion, microtubule regulation, cell adhesion, and intercellular signaling; it is essential for maintenance of T-tubule structure; important regulator of t-tubule membrane trafficking and Ca2^+^-dependent repair during stress/injury	No	Yes, DCM	Chase et al. (2009), Hofhuis et al. (2017), Hofhuis et al. (2020), Kerr et al. (2013), Nishikawa et al. (2016), Wenzel et al. (2007)
*SPEG*	Striated muscle preferentially expressed protein kinase (SPEG)	Myosin light chain kinase family protein important for cardiac development; it interacts with key proteins within the JMC (e.g. myotubularin 1, RyR2 and JPH2); it plays a critical role in the maintenance of JMC integrity and SR Ca^2+^ handling	No	Yes, DCM and non-compaction cardiomyopathy	(Agrawal et al. (2014), Quick et al. (2017), Wang et al. (2017)
*CSRP3*	Muscle LIM protein (MLP)	Essential nuclear regulator of myogenic differentiation; it stabilizes T-cap interaction with titin; MLP/T-cap/titin complex are thought to serve as a mechanical stress sensor	Yes	Yes, DCM	Arber et al. (1997), Bos et al. (2006), Geier et al. (2003), Knöll et al. (2002), Mohapatra et al. (2003), Vafiadaki et al. (2015)
*DMD*	Dystrophin	Cytoskeletal protein which provides a structural link between cytoskeleton and extracellular matrix promoting membrane stability and transduction of mechanical force from the extracellular matrix during muscle contraction/stretch; it localizes in both general sarcolemma and T-tubules	No	Yes, DCM	Kaprielian et al. (2000), Kawada et al. (2003), Lindner (1957), Mestroni et al. (2014)
*SYPL2*	Mitsugumin 29	Structural protein that participates in controlling the maturation and development of the T-tubule structure and the maintenance of intracellular Ca^2+^ signaling in skeletal muscle; in the heart it preserves T-tubule structure during failure serving as a brace to surround the T-tubule	No	Yes, DCM	Correll et al. (2017), Foster et al. (2016), Nishi et al. (1999), Xu et al. (2006)
*MTM1*	Myotubularin	Lipid phosphatase with putative role in T-tubule/SR network morphogenesis and/or remodeling	No	No	Al-Qusairi et al. (2009), Buj-Bello et al. (2008), Dowling et al. (2009)
*TRDN*	Triadin	Structural protein that links the calsequestrin (Casq2) to the SR ryanodine receptor Ca^2+^-release channels in the junctional SR	No	Yes, CPVT	Chopra and Knollmann (2013), Shen et al. (2007)

*SR* sarcoplasmic reticulum, *HCM* hypertrophic cardiomyopathy, *DCM* dilated cardiomyopathy, *JMC* junctional membrane complexes, *LTCC* L-type Ca^2+^ channels, *RyR* ryanodine receptors, *CPVT* Catecholaminergic Polymorphic Ventricular Tachycardia

As largely described above in common forms of “sarcomeric” HCM, T-tubular loss, when present, is not a direct result of the initial myofilament hit but rather is part of the ongoing process of electro-mechanical and structural remodeling that occurs in cardiomyocytes during the development of the disease. In the above-mentioned rare forms of cardiomyopathy, instead, the mutation affects genes coding for proteins mostly implicated in E–C coupling and membrane trafficking, tubule formation and maintenance. In such “non sarcomeric” HCM forms we can speculate that T-tubule remodeling may be a primary direct consequence of the mutation that drives the development of the disease.

However, this field of investigation has just started, and a lot of work is needed to determine the exact role of these proteins in T-tubule regulation and dysfunction, especially in human cardiac tissue.

As an example, recent studies have highlighted the crucial role of Junctophilin-2 (JPH2) in the correct assembly and maintenance of T-tubule-SR-Z disc connections (van Oort et al. 2011). JPH2 is a structural cardiac calcium handling protein, which physically approximates the cardiomyocyte T-tubules to the SR (Beavers et al. 2014; Takeshima et al. 2000). Decreased JPH2 expression was observed in human and animal models of hypertrophy and HF and has been linked to T-tubule remodeling (Frisk et al. 2016; Minamisawa et al. 2004; Wei et al. 2010; Xu et al. 2007). Moreover, inherited mutations in *JPH2* have been found in patients with both hypertrophic and dilated cardiomyopathy (Bongini et al. 2016; Jones et al. 2019; Landstrom et al. 2011; Matsushita et al. 2007). Specific cardiac knockout of JPH2 gene in transgenic mouse models leads to cardiomyocyte hypertrophy and abnormal intracellular calcium-handling, severe reduction of T-tubule density, orphaned and unregulated RyRs, and abnormal E–C coupling leading to global cardiac dysfunction (van Oort et al. 2011). Recent work provided further evidence of a crucial role of JPH2 in t-tubule structure maintenance. In particular, JPH2 overexpression has been indeed observed to restore T-tubule structure and normalize SR Ca^2+^ release in failing cardiomyocytes (Chen et al. 2012; Guo et al. 2015; Reynolds et al. 2016). All these observations suggest that JPH2 plays an important role in determining the physiological T-tubular structure, and that changes in its expression may be a primary determinant of T-system remodeling in “non-sarcomeric” forms of HCM.

## Conclusions

In conclusion, little is known about HCM-associated T-tubular remodeling in the “non-failing hypertrophic” stage of the disease (Fig. 2B, Table 1). The observation of reduced cell capacitance/cell volume ratio in HCM myocytes from myectomy samples is a strong indication of a disrupted T-tubular network (Fig. 4A) (Coppini et al. 2018), as observed in HF and “end-stage” HCM. However, both electron microscope (Maron et al. 1975a, b) and confocal microscope (Ferrantini et al. 2018) (Fig. 4B) studies, prompt us to imagine a more complex scenario, with large intra-myocardial variability in cell size and T-tubule architecture (Figs. 2B, 4B) and, potentially, T-tubule proliferation phenomena as described in animal models of compensated hypertrophy. The effects expected from a loss of T-tubules in terms of E–C coupling are mostly non-evident in HCM myocardium, “covered” by marked membrane current and calcium handling secondary remodeling processes, that occur downstream to the initial genetic-driven sarcomeric hit (Table 3). However, by comparing changes observed in HCM myocardium to those introduced by acute (experimentally-induced) detubulation, we highlight how the inhomogeneity and spatio-temporal dissynchrony of calcium activation, introduced by T-tubular disruption, could play a crucial role for the propensity towards arrhythmias and the slow force generation in HCM (Fig. 2A, Table 3). Finally, rare forms of “non sarcomeric” HCM have been described, associated to genes coding for proteins implicated in T-tubule formation and maintenance as well as E–C coupling or membrane trafficking. In such forms, T-tubule remodeling could occur as a primary direct consequence of the mutation and drive the development of the disease.

**Table 3:** Point-by-point comparison among acute detubulation, non-failing hypertrophic stage of HCM and terminal heart failure

	HCM non-failig hypertrophic	HF	Acute detubulation
Action potential duration	Prolonged*	Prolonged^#^	Shorthened
L type calcium current	Increased, slower inactivation	Unchanged or increased, unchanged or slower inactivation	Decreased, slower inactivation
Na^+^ current	Increased Late Na^+^ current	Unchanged or increased Late Na^+^ current	Unchanged
K^+^ currents	Decreased	Decreased	Unchanged
Spontaneous Ca waves	Increased	Increased	Decreased
Calcium transient amplitude	Modestly decreased or unchanged	Markedly decreased	Decreased
Calcium transient peak time	Prolonged	Prolonged	Prolonged
Calcium transient decay time	Prolonged	Prolonged	Modestly prolonged
Force-frequency relationship	Preserved	Impaired	Impaired
Twitch amplitude	Modestly decreased or unchanged	Markedly decreased	Decreased
Twitch peak time	Prolonged	Prolonged	Prolonged
Twitch decay time	Prolonged	Prolonged	Modestly prolonged
References	Coppini et al. (2013)	Lehnart et al. (2009), Coppini et al. (2013), Roe et al. (2015)	Kawai et al. (1999), Brette et al. (2002), Brette et al. (2005), Ferrantini et al. (2014)

Characteristics in terms of action potential, calcium transient and intact muscle contraction among the three different conditions
